# Characteristics of denitrification and microbial community in respect to various H_2_ pressures and distances to the gas supply end in H_2_-based MBfR

**DOI:** 10.3389/fmicb.2022.1023402

**Published:** 2022-09-23

**Authors:** Haixiang Li, Ruize Sun, Xuehong Zhang, Hua Lin, Yi Xie, Yu Han, Yongxing Pan, Dunqiu Wang, Kun Dong

**Affiliations:** College of Environmental Science and Engineering, Guilin University of Technology, Guilin, China

**Keywords:** denitrification, H_2_ pressure, microbial community, MBfR, nitrate reduction\keywordbelowspace-30pt

## Abstract

The hydrogen-based hollow fiber membrane biofilm reactor (H2-based MBfR) has shown to be a promising technology for nitrate (NO_3_^–^–N) reduction. Hollow fiber membranes (HFM) operating in a closed mode in an H_2_-based MBfR often suffer from reverse gas diffusion, taking up space for the effective gas substrate and resulting in a reduction in the HFM diffusion efficiency, which in turn affects denitrification performance. In this work, we developed a laboratory-scale H_2_-based MBfR, which operated in a closed mode to investigate the dynamics of denitrification performance and biofilm microbial community analysis at different H_2_ supply pressures. A faster formation of biofilm on the HFM and a shorter start-up period were found for a higher H_2_ supply pressure. An increase in the H_2_ pressure under 0.08 MPa could significantly promote denitrification, while a minor increase in denitrification was observed once the H_2_ pressure was over 0.08 MPa. Sequencing analysis of the biofilm concluded that (i) the dominant phylum-level bacteria in the reactor during the regulated hydrogen pressure phase were *Gammaproteobacteria* and *Alphaproteobacteria*; (ii) when the hydrogen pressure was 0.04–0.06 MPa, the dominant bacteria in the MBfR were mainly enriched on the hollow fiber membrane near the upper location (Gas inlet). With a gradual increase in the hydrogen pressure, the enrichment area of the dominant bacteria in MBfR gradually changed from the upper location to the distal end of the inlet. When the hydrogen pressure was 0.10 MPa, the dominant bacteria were mainly enriched on the hollow fiber membrane in the down location of the MBfR.

## Introduction

Nitrate is an oxidizing anion and worldwide an increase of nitrate contamination observed in surface water and groundwater has become an increasing problem in recent years, as a result of the excessive use of fertilizers and pesticides in agriculture, the uncontrolled discharge of industrial and domestic wastewater and the intensive use of fertilizers in agriculture (Karanasios et al., 2010; Dong et al., 2022; Jiang et al., 2022), while soils have high mineral solubility and low water-holding capacity, making it easy for nitrate to infiltrate into groundwater with rainwater or farm irrigation, cause irreversible nitrate pollution. Nitrate contamination of surface water and groundwater not only endangers ecological environment, but also poses a serious threat to ecological safety and human health. Meanwhile, high nitrate concentrations in drinking water can trigger the production of some carcinogenic nitrosamines in some water bodies, which can cause methemoglobinemia (also known as “blue baby syndrome”), among other adverse health effects (Fossen Johnson, 2019). The World Health Organization (WHO) and US Environmental Protection Agency (USEPA) suggested 11.3 or 10 mg/L as a treat goal for the maximum contaminant level (MCL) of nitrate in potable water. The European Community recommended its drinking water action level was 5.6 mg NO_3_^–^–N/L (Lee and Rittmann, 2000).

NO_3_^–^–N cannot be easily removed by conventional physical–chemical water treatment and advanced processes, such as ion change, chemical reduction, electrodialysis, reverse osmosis, or catalytic denitrification reduction (Della Rocca et al., 2007; Demirel and Bayhan, 2013). Problems associated with the abovementioned technologies include high capital and energy costs, potential secondary pollution problems, and the production of large quantities of waste brine containing high concentrations of nitrate. Fortunately, nitrate can be reduced to nitrogen by denitrifying bacteria (DNB), which can use nitrate as a terminal electron acceptor for growth under anoxic conditions (Xia et al., 2015).

As an emerging nitrate removal technology that originated from membrane technology, hydrogen autotrophic denitrification can be used to remove nitrate from groundwater and surface water (Keisar et al., 2021) and has shown to be preferable over conventional treatment strategies, such as physicochemical and heterotrophic denitrification treatments. In addition, in H_2_-based hollow fiber membrane biofilm reactor (MBfR), electron donors and acceptors diffuse into the biofilm from different sides (inverse diffusion). The driving force for the mass transfer of H_2_ in the biofilm has shown to be the concentration gradient formed by the hydrogen consumption of the bio-reduction process in the biofilm (Xia et al., 2015). The increased H_2_ utilization (close to 100%) can avoid the risk of reactor explosion due to H_2_ accumulation (Long et al., 2020). Meanwhile, H_2_ has shown to be an excellent electron donor for autotrophic denitrification, and its advantages include a lower unit cost source of electrons, elimination of the need for organic C sources, non-toxicity to humans, and the production of significantly less excess biomass. Autotrophic denitrification systems using hydrogen as an electron donor produce 50% less biosolids than heterotrophic denitrification systems using organic matter as an electron donor. In addition, H_2_-based MBfR based on hydrogen autotrophic denitrification has shown to be an ideal option for groundwater and surface water remediation due to high NO_3_−−N removal, sound H_2_ utilization efficiency, no subsequent contamination, low biomass production, a low operating cost, and the ability to compensate for the low availability of carbon sources in groundwater (Lee and Rittmann, 2002; Karanasios et al., 2010; Keisar et al., 2021).

**FIGURE 1:**
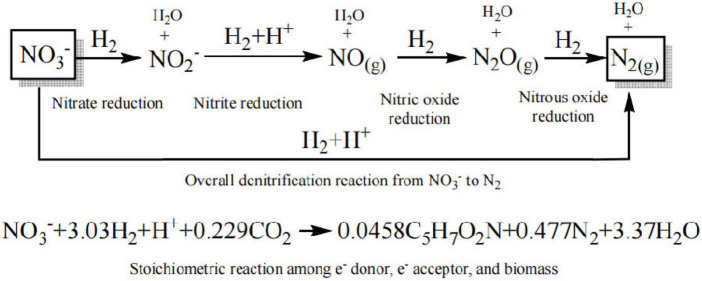
Stoichiometric relationship between H_2_/NO_3_ and the biomass.

In H_2_-based MBfR, pressurized H_2_ is supplied within the lumen of the hollow fiber membrane (HFM), and the gaseous substrate is then diffused anisotropically in a bubble-free manner through the microporous membrane walls to the microorganisms living on the walls of the bubbleless gas-transfer membrane, forming a biofilm on the surface of the HFM. The reaction stoichiometry in the denitrification process is illustrated in Figure 1, in which H_2_ serves as the electron donor, nitrate as the electron acceptor, which is converted to nitrogen gas, and CO_2_ as the carbon source for the autotrophic microorganisms (Lee and Rittmann, 2002; Xia et al., 2016).

During the operation of an H_2_-based MBfR, a variety of conditions and environmental elements have an effect on the removal of contaminants. These influences include the influent flow rate, influent contaminant concentration, pH, temperature, and hydrogen pressure, with hydrogen pressure the most significant factor. This is because hydrogen provides an electron donor for the reaction, and the hydrogen pressure can be adjusted quickly and efficiently in the H_2_-based MBfR system (Rittmann et al., 2004; Long et al., 2020). For example, Xia et al. (2011) operated an H_2_-based MBfR to remove 2-chlorophenol from groundwater and found that the removal rate was nearly 99% when the hydrogen pressure increased from 0.02 to 0.05 MPa; however, the hydrogen pressure was not a limiting factor for the removal of 2-chlorophenol, and a sufficient hydrogen pressure could ensure the active thickness of the biofilm and minimize the accumulation of excessive biofilm, in which H_2_ was the primary electron donor, thus facilitating the degradation of 2-chlorophenol. Xia et al. (2010) also showed that with increasing H_2_ pressure, the total nitrogen removal rate reached more than 97% when the nitrate load was increased from 0.17 to 0.34 g NO_3_^–^-N/ (m^2^day). Chung et al. (2006a) showed the effect of modulating the hydrogen pressure on the removal of selenate, and as the H_2_ pressure increased from 2.5 to 5.5 psi, the Se (VI) flux normalized by its effluent concentration increased from 912 to 13,500 m/d. This suggested that selenate reduction was largely dependent on the increase in hydrogen pressure, and the hydrogen pressure had some effect on the removal of pollutants in MBfR.

**FIGURE 2:**
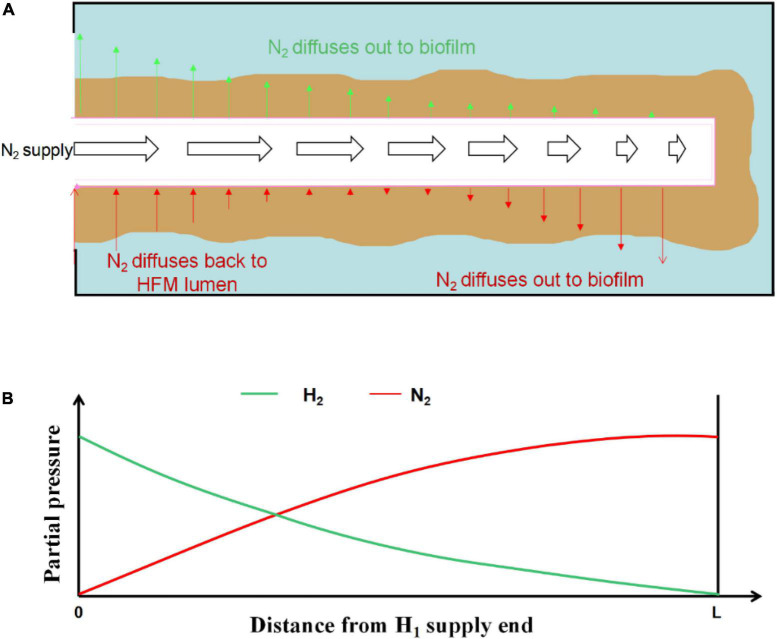
Pressurized H_2_ supply and N_2_ back diffusion in a sealed end hollow fiber membrane (HFM): **(A)** Schematic of a single HFM with biofilm attached; **(B)** H_2_ and N_2_ partial pressure in the HFM lumen.

Most of the H_2_-based MBfRs were operated in closed-end mode, because this allowed for high gas transfer efficiency (Perez-Calleja et al., 2017). Thus, 100% transfer efficiency of H_2_ could be easily achieved if the system was designed and managed properly, thus preventing H_2_ bubble formation in the reactor and the possibility of an explosive environment (Lee and Rittmann, 2003). The closed-end operation mode also minimized the operating cost of electron donor usage by avoiding gas waste (Lee and Rittmann, 2003). Aybar et al. (2014) reported that the closed-end operating mode in the O_2_-based MBfR could reduce energy costs by more than 85% compared with the conventional activated sludge process. However, despite the high gas transfer efficiency in the closed-end HFM, previous research revealed a negative influence on the gas transfer rate caused by gas back-diffusion (Perez-Calleja et al., 2017). Gas back-diffusion can be defined as other dissolved gases in the biofilm, mostly N_2_, which inevitably diffuses back into the HFM lumen (Ahmed and Semmens, 1992; Perez-Calleja et al., 2017). In addition, the production of N_2_ in the biofilm of H_2_-based MBfR during the denitrification process could enhance the effect of back-diffusion. As shown in Figure 2B, with an increase in the distance from the H_2_ supply end, the partial pressure of H_2_ in the lumen of HFM gradually decreases, with the opposite trend for N_2_ partial pressure. N_2_ may diffuse out with a higher flux than H_2_ from the HFM to the biofilm (Figure 2A) as the N_2_ partial pressure moves higher close to the distal end (Figure 2B). In the sealed gas supply mode of the H_2_-based MBfR, the diffusion flux of the effective gas at the end of the HFM to the biofilm is usually low, which can prevent many fiber membranes from operating and decreasing the utilization efficiency, ensuing in the waste of the membrane material and reactor volume. Jiang et al. (2020) also showed that most of the functional genes associated with the microbial metabolism and denitrification processes in microbial communities on HFM in closed operation mode showed a weakening trend as they moved away from the gas supply side, under the influence of gas counter-diffusion. Therefore, this back-diffusion reduced the total effectiveness of the biofilm and the average denitrification efficiency. Although the negative influence of back-diffusion on the performance of gas-based MBfRs has been acknowledged, exactly how back-diffusion affects the microbial community structure in the biofilm and the denitrification performance of the reactor under various H_2_ supplying pressures in H_2_-based MBfR is yet to be elucidated. Therefore, research is needed to address this knowledge gap.

## Materials and methods

### Experimental setup and operating conditions

**FIGURE 3:**
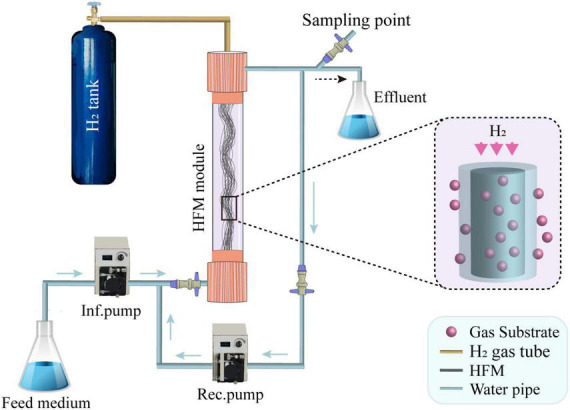
A schematic of the H_2_-based hollow fiber membrane biofilm reactor (MBfR) set-up, where Inf. and Rec. indicate the influent and recirculation, respectively, and HFM is the hollow fiber membrane.

A schematic of the lab-scale H_2_-based MBfR used in this study is shown in Figure 3, and the physical characteristics of the reactor are listed in Table 1. The H_2_-based MBfR consisted of an HFM module with 20 HFMs located inside of a vertical plexiglass cylindrical shell, where the interior of the HFM module was connected to a pressurized H_2_ supply at one end and sealed at the other end (Chung et al., 2006b). The HFMs were constructed from polyvinyl chloride (PVC) (Watercode, Guangzhou, China), with a pore size of 0.1 μm, which were used in the reactor to deliver the bubbleless H_2_ through the HFM wall, and H_2_ diffused from the lumen of the HFM through the wall and into biofilm, where it was oxidized. The top of the HFM module was connected to an ultrapure H_2_ tank to supply the pressurized H_2_, and the bottom was sealed with epoxy glue. The H_2_ supplied pressure could also be adjusted with a gas regulator on the H_2_ tank. The HFM provided an adhesion environment for the microorganisms, and hydrogen diffused from inside the membrane to the outside by microporous aeration, providing an electron donor for the microorganisms attached to the HFM.

**TABLE 1:** Physical characteristics of the MBfR system.

Parameter	Units	Value
MBfR height	cm	50
Inner diameter	mm	45
Number of HFM		20
Active length of HFM	cm	45
HFM inner diameter	mm	1.0
HFM outer diameter	mm	1.5
HFM pore size	μm	0.02
Active surface area	m^2^	0.06
Active volume	L	0.6

A single peristaltic manifold (BT101L-DG-1, Lead Fluid, China) was used with PVC tubing to provide a synthetic (see Section “Synthetic influent” for details) medium-feed rate of 1 mL/min into the H_2_-based MBfR from the bottom, resulting in an hydraulic retention time (HRT) of 10 h. A recirculation pump (BT101L-YZ15/25, Lead Fluid, China) was used to provide a high recirculation ratio (100:1) and flowrate of 100 ml/min, to promote a completely mixed condition in the H_2_-based MBfR. The effluent was located on the upper part of the reactor, and the aquatic samples were collected from the effluent pipe.

### Synthetic influent

The synthetic feed medium was prepared from tap water amended with NaNO_3_ (30 mg/L as nitrogen) and 80 mg/L NaHCO_3_ as the inorganic carbon source, as well as trace mineral elements. The composition of trace mineral elements was the same as in our previous study (Li et al., 2018): 1,000, CaCl_2_⋅2H_2_O; 1,000, FeSO_4_⋅7H_2_O; 13, ZnSO_4_⋅7H_2_O; 4, MnCl_2_⋅4H_2_O; 38, H_3_BO_3_; 25, CoCl_2_⋅6H_2_O; 1, CuCl_2_⋅2H_2_O; 1, NiCl_2_⋅6H_2_O; 4, Na_2_MoO_4_⋅2H_2_O; and 4, Na_2_SeO_3_ (μg/L). The pH of the bulk liquid was balanced to around 7.5 with phosphate buffer (216 mg/L Na_2_HPO_4_⋅12H_2_O + 236 mg/L KH_2_PO_4_) to avoid a sharp increase in pH during the denitrification process. All synthetic feed media were purged with N_2_ to remove the dissolved O_2_ in the influent. The pH of the bulk liquid was balanced to around 7.5 with phosphate buffer (216 mg/L Na_2_HPO_4_⋅12H_2_O + 236 mg/L KH_2_PO_4_) to avoid a sharp increase in pH during the denitrification process.

### Biofilm structural analysis

Four parallel H_2_-based MBfRs were used in this study, and the start-up conditions for each reactor were conducted under the same procedures and operating conditions when H_2_ was supplied to the HFMs. The inoculation of seed biomass was obtained from other denitrifying H_2_-based MBfRs in our group. After successful start-up, H_2_ supplying pressures of 0.04, 0.06, 0.08, and 0.1 MPa were set for each reactor, and all bulk liquid was replaced with fresh medium immediately. Other than the H_2_ supply pressure, the other operating conditions were the same in the four reactors as follows: influent NO_3_^–^ = 30 mg N/L, HRT = 10 h, and pH = 7.5.

### Sampling and analytical methods

#### Aquatic sample

The NO_3_^–^-N removal flux was calculated by Eq. 1 (Lai et al., 2014; Zhao et al., 2014), and the reactor order was also estimated to investigate the effect of H_2_ pressure change on the denitrification performance of the reactor, according to Eq. 2 (Li et al., 2013):


(1) $$\text{J}=\frac{\text{Q}}{\text{A}}\left({\text{S}}_{inf}-{\text{S}}_{eff}\right)$$

(2) $${\text{k}}^{{}^{\prime }}=\frac{\text{d}\left(\text{l}g\text{J}\right)}{\text{d}\left(\text{l}g{\text{S}}_{eff}\right)}$$

where *J* is the removal flux (g/(m^2^⋅d)), *Q* is the influent flow rate (m^3^/d), *A* is the membrane surface area (m^2^), and *S*_*inf*_ and *S*_*eff*_ are the influent and effluent NO_3_^–^-N concentrations, respectively (g/m^3^).

#### Biofilm sampling and high-throughput sequencing

For each reactor, samples were collected from three different distances of 5, 20, and 35 cm from the H_2_ supply end, denoted as the upper, middle, and down, respectively. The sample collection procedure was as follows: (i) we selected three HFMs from each reactor, (ii) we then cut off three 2-3 cm sections from the HFM segments at the three different locations described above, (iii) we combined the three segments into one sample for each location, and (iv) we detached the biofilm from the HFMs using an ultrasonic instrument (SK3300-35 kHz, China). The collected samples were then stored at −80°C until further processing.

The collected biofilm samples were sent out, and we analyzed the bacterial species by high-throughput sequencing at Novogene Co., Ltd. (Suzhou, China) for microbial structural analysis. The 16S rRNA gene sequence libraries were then prepared using polymerase chain reaction (PCR) with universal primers, and the DNA was PCR amplified with 341F (CCTACGGGNGGCWGCAG) and 805R (GACTACHVGGGTATCTAATCC) of the V3–V4 hypervariable gene regions.

## Results and discussion

### Effects of electron donor on the denitrification performance

After 60 days of inoculation, a complete NO_3_^–^–N removal was achieved under an HRT of 10 h with an influent concentration of 10 mg N/L. The NO_3_^–^–N concentration of the H_2_-based MBfR effluent was basically stable at about 0.10 mg/L for more than 3 days without obvious fluctuations, and the final removal rate of the H_2_-based MBfR reached 99.00%, while a yellow-brown biofilm appeared on the surface of the HFM. This indicated that the H_2_-based MBfR hanging membrane domestication stage was complete, and based upon this, start-up of the H_2_-based MBfR was considered successful.

**FIGURE 4:**
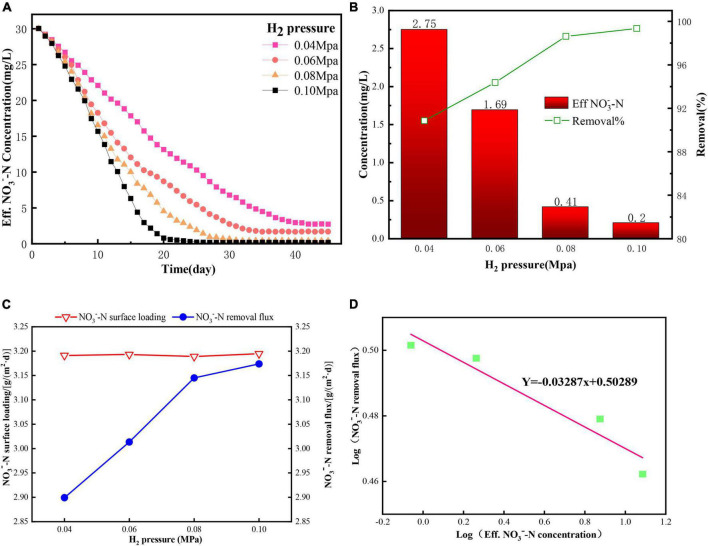
The effect of H_2_ supply pressure on the denitrification performance of the H_2_-based MBfR: **(A)** The NO_3_^–^–N concentrations in the effluent from the replacement of the bulk liquid with 30 mg N/L, **(B)** Effluent concentrations and removal percentages of NO_3_^–^–N under steady states, **(C)** Surface loading and removal flux, and **(D)** Reaction order of NO_3_^–^–N removal under the four H_2_ supply pressures of 0.04, 0.06, 0.08, and 0.1 MPa operating in the four reactors.

Hydrogen serves as an electron donor for microorganisms, and the effect of variations in the hydrogen pressure on the NO_3_^–^–N removal effect is not negligible (Nerenberg and Rittmann, 2004). To evaluate the effects of H_2_ supply pressure on denitrification performance of H_2_-based MBfR, pressures of 0.04, 0.06, 0.08, and 0.1 MPa were carried out in four separate reactors, and the results are shown in Figure 4. During this phase of the experiments, all of the bulk liquids were replaced with fresh medium containing 30 mg NO_3_^–^–N/L in each reactor on day 1, and then the reactors were operated in continuous mode with an influent flowrate of 1 mL/min. As depicted in Figure 4A, the reactor reached a steady state (no more changes in the effluent concentration), which was faster with higher H_2_ supply pressure. This required 25 days for an H_2_ pressure of 0.1 MPa, 30 days for 0.08 MPa, 35 days for 0.06 MPa, and 42 days for 0.04 MPa. These results indicated that a higher availability of electron donors in the biofilm could shorten the stabilization time of the H_2_-based MBfR. Previous studies reported that hydrogen availability had a significant influence on the removal rate and biofilm growth (Karanasios et al., 2010); therefore, higher hydrogen availability could lead to faster biofilm growth and a shorter time to reach a steady state.

H_2_ was the only electron donor supplied to the system. Thus, H_2_ availability in the reactor would directly affect the NO_3_^–^–N reduction performance. Lee and Rittmann (2002) verified that the H_2_ supply pressure was the most important factor in controlling the denitrification efficiency. Thus, 100% NO_3_^–^–N would be possible if the H_2_ pressure was sufficiently high (Lee and Rittmann, 2002; Xia et al., 2010). Figure 4B shows the effluent concentrations and removal percentage of NO_3_^–^–N at a steady state with various H_2_ supply pressures. When the H_2_ pressure increased from 0.04 to 0.1 MPa, there was a non-linear decrease in the NO_3_^–^–N concentration in the effluent from 2.75 to 0.20 mg/L. The NO_3_^–^–N removal performance in the removal percentage (Figure 4B) and flux (Figure 4C) showed a linear increase from 90.8% and 2.90 g/(m^2^⋅d) to 98.6% and 3.14 g/(m^2^⋅d), respectively, as the H_2_ pressure increased from 0.04 to 0.08 MPa, and then showed a slight increase when the H_2_ pressure further increased to 0.1 MPa, and these results were similar to Ziv-El and Rittmann (2009). Although the highest removal percentage of 99.3% was achieved, an excessively high H_2_ supply pressure possibly caused an off-gassing problem, which created an explosive environment. Lee and Rittmann (2002) recommended that the H_2_ concentration in the liquid-phase/bulk liquid should be less than 9 μg/L in H_2_-MBfR, for safety reasons. In addition, the NO_3_^–^–N concentration was higher than the half-maximum-rate concentration for DNB (1.02 mg/L) when the H_2_ pressure was higher than 0.06 MPa, indicating that NO_3_^–^–N availability was a limiting factor for denitrification (Tang et al., 2012). Therefore, an H_2_ supply pressure of less than 0.08 MPa was suggested with an influent containing NO_3_^–^–N 30 mg/L at an HRT of 10 h for the H_2_-based MBfR in this study.

To estimate the reaction order to investigate the influence level of H_2_ supply pressure on nitrate removal, the log (effluent NO_3_^–^–N removal flux) versus log (effluent NO_3_^–^–N concentration) was plotted, as shown in Figure 4D. The fitted slope was negative (−0.03), which meant that the removal flux increased with decreasing NO_3_^–^–N concentration, indicating that denitrification was strongly dependent on the H_2_ pressure.

### Relative abundance analysis of the biofilm microbial community

**FIGURE 5:**
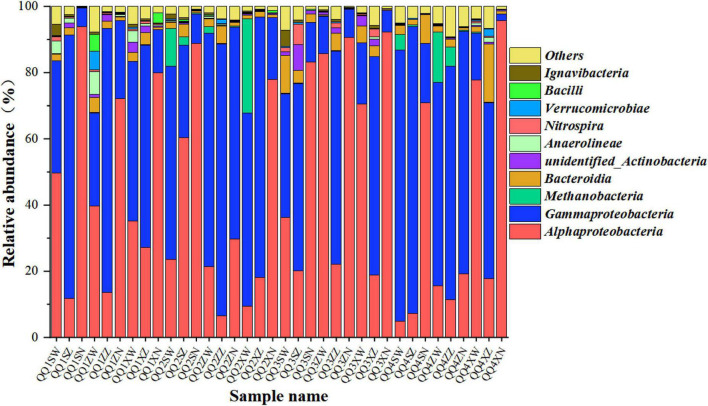
A class-level relative abundance of top 10 dominant bacteria of pyrosequences from biofilm samples taken in the H_2_-based MBfR under the four phases operating with H_2_ supplying pressure, where “H1,” “H2,” “H3,” and “H4” represent H_2_ pressures of 0.04, 0.06, 0.08, and 0.1, respectively; “U,” “M,” and “D” (the third letter of the sample name) represent the samples were obtained from the locations of upper, middle, and lower portions of the hollow fiber membranes (HFMs), and “O,” “D,” and “I” (the fourth letter of the sample name) denote the outer, middle, and inner layers of each location of the HFMs. The same labeling is used in Figure 6.

Figure 5 shows the taxonomy of the biofilm microbial communities for the H_2_-based MBfR under the four phases operating with H_2_ supply pressure. The H_2_ pressures of 0.04, 0.06, 0.08, and 0.1 MPa were labeled as “H1,” “H2,” “H3,” and “H4,” respectively, where “U,” “M,” and “D” (the third letter of the sample name) represent that samples obtained from the locations of the upper, middle, and down portions of the HFMs, and “O,” “D,” and “I” (the fourth letter in the sample name) denote the outer, middle, and inner layers of each location of the HFMs, respectively. For example, “H1UO” indicates that the sample was taken from the outer layers in the upper position of the HFM operating at an H_2_ supply pressure of 0.04 MPa.

As illustrated in Figure 5, *Alphaproteobacteria* and *Gammaproteobacteria* were the dominant bacteria in all biofilm samples at the class level. Many previous studies have reported that *Proteobacteria* play an important role in the biogradation of various contaminants in the H_2_-based MBfR and usually are the most abundant microorganisms (Lai et al., 2014; Park et al., 2016; Li et al., 2018). A small amount of *Methanobacteria* was also observed, indicating that methanogenesis was driven by the reaction of H_2_ and CO_2_, although it is considered to be undesirable metabolism. The presence of methane-producing bacteria in the H_2_-based MBfR was consistent with the results reported in previous studies (Martin et al., 2013, 2015).

**FIGURE 6:**
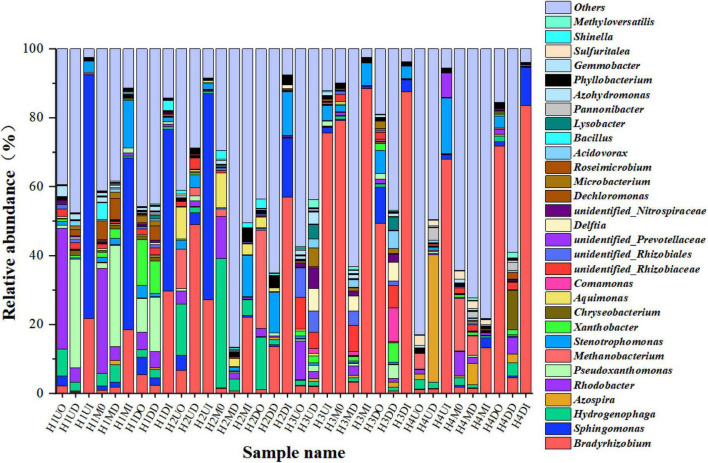
A genus-level relative abundance of the top 30 dominant bacteria of pyrosequences.

Because the dominant *Proteobacteria* contain a variety of functional bacteria and not all *Proteobacteria* can degrade nitrate, the biofilm microbial community was further analyzed and classified at the genus level, as shown in Figure 6. The bacterial community structures of all biofilm samples at the genus level were all very complex. Some well-known autotrophic DNB reported in the literature include *Hydrogenophaga, Rhodobacter, Rhodopseudomonas, Comamonas, Dechloromonas, Bacillus, Methyloversatilis, Achromobacter, Denitratisoma*, and *Pseudomonas* (Vasiliadou et al., 2006; Zhang et al., 2009; Zhao et al., 2011; Park et al., 2016; Wu et al., 2018), while *Bradyrhizobium* (Bedmar et al., 2005; Sánchez et al., 2011), *Azospira* (Byrne-Bailey and Coates, 2012; Rossi et al., 2015), and *Sphingomonas* (Wang and Skipper, 2004; Zhang et al., 2013; Garcia-Romero et al., 2016) have also been reported as nitrate biodegradation bacteria.

According to the distribution of the dominant species abundance location levels, as shown in Figure 6, *Bradyrhizobium* and *Sphingomonas* were considered to be the dominant species under hydrogen pressure-regulated phase, their average relative abundance were 24.89 and 8.38%, respectively. There were also eight genera with relative abundances above 1%, *Rhodobacter* (4.05%), *Hydrogenophaga* (3.47%), *Stenotrophomonas* (3.37%), *Pseudoxanthomonas* (2.99%), *Methanobacterium* (2.04%), *unidentified_Rhizobiaceae* (1.60%), *Azospira* (1.55%), and *Xanthobacter* (1.34%). The inner biofilm (the fourth letter of the sample name was I) on the HFM at the genus level was still the dominant species in abundance. The second highest abundance of *Bradyrhizobium* and *Sphingomonas* was in the location of the outer biofilm (the fourth letter of the sample name was O), which was the furthest from the HFM, and the third rank in abundance was located in the middle of the biofilm (the fourth letter of the sample name was D). With a gradual increase in the hydrogen pressure, the abundance distribution of the dominant strains on the inner layer also showed a corresponding increase. In terms of the position, under a hydrogen pressure of 0.04 MPa, the abundance distribution pattern consisted of the upper locations on the HFMs > the down locations on the HFMs > the middle locations of the HFMs under a condition of 0.04 MPa hydrogen pressure. At a hydrogen pressure of 0.04 MPa, *Sphingomonas* played a dominant role, and the abundance of *Sphingomonas* gradually decreased with increasing hydrogen pressure, while the location distribution pattern of the abundance of *Sphingomonas* at 0.06 MPa, which was consistent with 0.04 MPa, remained the same. When the hydrogen pressure continued to increase, the dominant strain *Bradyrhizobium* abundance distribution pattern gradually overstepped to the middle location between the proximal and distal ends, and finally enriched to the down locations of MBfR. In conclusion, the overall enrichment pattern of MBfR was that at a lower hydrogen pressure (0.04–0.06 MPa), the dominant strain was enriched on the upper location of the MBfR near the hydrogen port. As the hydrogen pressure increased, the enrichment area was in the middle position between the near and distal ends (0.08 MPa). At a hydrogen pressure of 0.10 MPa, the enrichment area on the HFM was found at the upper locations of MBfR, and its dominant strain enrichment pattern was consistent with the hydrogen pressure-regulated phase at the class level.

## Conclusion

A higher H_2_ supply pressure in the H_2_-based MBfR could promote the formation of a biofilm on the HFM and shorten the start-up period, resulting in various levels of effects on the denitrification performance in the steady state of the reactor. An increasing H_2_ pressure that did not exceed 0.08 MPa could significantly improve the denitrification performance, and once the H_2_ pressure exceeded 0.08, a minor increase in denitrification was observed. Through sequencing analysis of the biofilm at different locations from the H_2_ supply end in the H_2_-based MBfR operating under the pressure range of 0.04–0.1 MPa, we concluded that (i) *Bradyrhizobium* and *Sphingomonas* were dominant in the closest biofilm to the H_2_ supply end and became less competitive along the distance from the H_2_ supply end when the H_2_ pressure was lower than 0.08 MPa; therefore, there was less contribution to the total NO_3_^–^–N removal of the farther biofilm in H_2_-based MBfR; (ii) increasing the H_2_ supply pressure helped to enhance the average *Bradyrhizobium* abundance in the reactor, while extravagant H_2_ pressure (greater than 0.06 MPa in this study) possibly had little positive influence on the denitrification because of the NO_3_^–^–N limitation, but the safety risk could increase.

## Data availability statement

The raw data supporting the conclusions of this article will be made available by the authors, without undue reservation.

## Author contributions

KD, YP, YX, and HaL: conceptualization and methodology. RS, KD, YX, and YH: project administration and data curation. RS and KD: writing–original draft. KD and YP: writing–review and editing. KD, XZ, HuL, DW, and HaL: supervision. All authors contributed to the article and approved the submitted version.
